# From Repair to Prevention: The Emerging Role of Preventive Orthopedics

**DOI:** 10.7759/cureus.100008

**Published:** 2025-12-24

**Authors:** Hiram E Luigi Martinez, José Pablo Bibiloni Lugo, Leslian Vélez-Ramos, Ammar N Saigal, Rafael Señeriz Ortiz

**Affiliations:** 1 Department of Orthopaedic Surgery, Ponce Health Sciences University, Ponce, PRI; 2 Department of Family Medicine, Trinity Health, Minot, USA

**Keywords:** exercise therapy, fracture liaison services, injury prevention, musculoskeletal disorders, osteoarthritis, prehabilitation, preventive orthopedics

## Abstract

Musculoskeletal (MSK) conditions are leading causes of global disability and major drivers of healthcare utilization, surgeries, and productivity loss, with low back pain, osteoarthritis, and fragility fractures contributing most to the burden and expected to rise due to aging, obesity, and sedentary lifestyles. Work-related MSK disorders remain highly prevalent and are costly. This narrative review aimed to synthesize current preventive orthopedic strategies across the life course. Evidence was drawn from systematic reviews, meta-analyses, clinical guidelines, economic evaluations, and targeted searches, following best-practice standards for transparent narrative reviews. Preventive orthopedics integrates risk stratification; neuromuscular and ergonomic interventions; lifestyle-first osteoarthritis management; fall and fracture prevention, including osteoporosis screening and fracture liaison services; workplace MSK programs; perioperative prehabilitation; digital health technologies; and emerging pharmacologic strategies such as glucagon-like peptide-1 (GLP-1)-based weight management. These interventions demonstrate reductions in injury rates, improvements in pain and function, and meaningful health system efficiencies. Persistent challenges include adherence, implementation fidelity, reporting quality, and equitable access. Future priorities include pragmatic and equity-focused trials and clearer operationalization of prevention across musculoskeletal care pathways.

## Introduction and background

Musculoskeletal (MSK) conditions are the leading contributors to years lived with disability globally and a growing threat to healthy aging [1-3]. Low back pain is the single largest cause of disability worldwide, with substantial projected increases in prevalence and associated disability through 2050 [1]. Other MSK disorders, including osteoarthritis, neck pain, and regional pain syndromes, account for a large and rising share of disability across regions and income levels [2]. The World Health Organization documents emphasize that MSK conditions commonly restrict mobility and function, limit participation in work and social roles, and frequently coexist with cardiometabolic and mental health conditions [3].

The societal and economic consequences are substantial. In many countries, MSK complaints are among the most common reasons for primary care visits and sickness absence and are a major cause of disability pension [3-6]. Work related MSK disorders remain prevalent despite decades of ergonomic research, particularly in physically demanding occupations such as health care, agriculture, and construction [4-9].

Orthopedics has traditionally focused on structural repair, including fracture fixation, ligament reconstruction, arthroplasty, and spinal surgery. These procedures deliver large improvements in pain and function for many patients, yet a reactive, procedure centric model is poorly aligned with the chronic, recurrent nature of most MSK conditions. The emerging concept of preventive orthopedics reframes orthopedic practice as an integrated set of primary, secondary, and tertiary prevention strategies that seek to prevent incident MSK injury and disease, limit progression or recurrence, and reduce disability and complications after injury or surgery [10-13].

Primary prevention aims to prevent the first occurrence of injury or disease, for example through neuromuscular training in youth sport, ergonomics in high-risk work, and lifestyle measures to prevent osteoarthritis and osteoporosis [7-9,14-24]. Secondary prevention targets early or recurrent disease, such as structured programs to prevent low back pain recurrences and coordinated secondary fracture prevention via a fracture liaison service (FLS) [25-33]. Tertiary prevention focuses on minimizing disability and optimizing outcomes in established conditions, exemplified by perioperative prehabilitation and long-term post fracture secondary prevention [27,34-40]. Conceptual and scoping work across MSK disciplines underscores the need to operationalize these levels of prevention more precisely in orthopedic practice [11-13].

Building on an integrative scoping review of 40 systematic reviews that defined a preventive orthopedics paradigm [11-13], this review aims to summarize current trends in preventive orthopedics across the life course, describe clinical and health system benefits of preventive strategies, identify major barriers and implementation challenges, and propose future directions and a research agenda to advance preventive orthopedics within routine MSK care.

## Review

Methods

Design and Methodological Framework

This review was conducted as a narrative synthesis designed to integrate heterogeneous evidence across preventive interventions, populations, and care settings. Narrative reviews are appropriate when evidence spans diverse conditions and intervention types that cannot be meaningfully pooled in a meta-analysis. To enhance transparency and methodological rigor, we followed the Scale for the Assessment of Narrative Review Articles (SANRA) criteria for high-quality narrative reviews [41]. Although this was not a systematic review, selected Preferred Reporting Items for Systematic Reviews and Meta-Analyses (PRISMA)-informed reporting elements were incorporated to strengthen transparency regarding the search process, evidence sources, screening considerations, and overall flow of evidence [42].

Evidence Sources

The evidence base was drawn from two complementary categories: foundational scoping and systematic reviews, and additional targeted literature addressing recent advances and emerging domains in preventive orthopedics.

Foundational evidence was identified through exploratory searches and citation tracking of key scoping and systematic reviews describing preventive orthopedics across sport, occupational, community, and clinical MSK domains (e.g., [11-16,24]). These reviews established core concepts, intervention categories, and methodological frameworks that informed the structure and scope of the present synthesis. 

To update and expand upon these foundational sources, additional targeted literature was identified focusing on priority areas relevant to contemporary preventive orthopedics.

Targeted Search Strategy

Targeted searches were conducted in PubMed covering the period from January 2010 through November 2025. Search terms and combinations were selected to capture key domains within preventive orthopedics and implementation science. These included terms related to MSK conditions and prevention (e.g., MSK, injury prevention, neuromuscular training, anterior cruciate ligament (ACL) injury, fall prevention, fracture liaison service, osteoporosis screening, dual-energy X-ray absorptiometry (DEXA), ergonomic intervention, low back pain prevention, prehabilitation), as well as digital and technological approaches (digital MSK, telehealth MSK, machine learning osteoarthritis, deep learning osteoarthritis, smartphone gait analysis), pharmacologic prevention (glucagon-like peptide-1 or GLP-1, semaglutide, incretin therapy, weight management in osteoarthritis), and implementation science frameworks (implementation science, Consolidated Framework for Implementation Research (CFIR), and Reach, Effectiveness, Adoption, Implementation, and Maintenance Framework (RE-AIM)).

Searches were supplemented by citation tracking of key reviews and landmark trials. Reporting guidance followed SANRA criteria for narrative reviews, alongside intervention description frameworks including Template for Intervention Description and Replication (TIDieR) and TIDieR-Rehab [41,43,44].

Study Selection and Screening (PRISMA-Inspired)

Eligible evidence was identified through iterative screening of titles, abstracts, and full texts. Inclusion focused on empirical studies, clinical guidelines, and high-quality reviews relevant to preventive orthopedics. Included evidence comprised scoping and systematic reviews [11-16,24]; randomized or quasi-randomized trials, including landmark secondary prevention trials such as the WalkBack Trial [30]; clinical practice guidelines, including United States Preventive Services Task Force (USPSTF) 2024-2025 screening recommendations [25,26] and American College of Rheumatology/Osteoarthritis Research Society International (ACR/OARSI) osteoarthritis guidelines [17-19]; economic modeling and cost-effectiveness analyses [33-34]; implementation science studies and frameworks, including CFIR and RE-AIM [45-47]; validated digital, wearable, and artificial intelligence-based studies, including biomechanical wearables and smartphone-based fall-risk assessment tools [48-52]; machine-learning applications in osteoarthritis addressing early detection and disease progression [53-55]; and emerging pharmacologic prevention strategies, including GLP-1 receptor agonists and dual incretin therapies relevant to knee osteoarthritis [56-58].

Excluded Evidence

Studies were excluded if they focused primarily on surgical techniques or operative management, addressed non-MSK or unrelated medical conditions, consisted of commentaries, expert opinions, or conceptual papers lacking empirical data, or were published in languages other than English.

Terminology and Scope

Standard definitions of primary, secondary, and tertiary prevention were applied, consistent with recent MSK prevention reviews and public health frameworks [10-13]. Chronic MSK pain was conceptualized both as a disease entity and as a common downstream consequence of MSK injury and degenerative conditions [11,12,59]. For the purposes of this review, orthopedic prevention was interpreted broadly to include surgeon-led, multidisciplinary, and community-based strategies when interventions targeted MSK outcomes directly relevant to orthopedic practice.

Flow of Evidence (Simplified PRISMA-Like Summary)

The evidence base was assembled using two complementary pathways. First, core scoping and systematic reviews were identified through exploratory searching and citation tracking [11-13,24]. Second, targeted PubMed searches identified approximately 1,100 records, from which 186 titles were screened, 112 abstracts were reviewed, and 74 full texts were assessed. A total of 56 studies were ultimately included, encompassing clinical guidelines, randomized and quasi-randomized trials, economic analyses, implementation evaluations, digital and wearable technologies, and machine-learning studies. This streamlined approach enhanced transparency regarding evidence identification and selection while remaining consistent with the goals of a narrative synthesis rather than a full systematic review.

Conceptual foundations of preventive orthopedics

Life Course Perspective

MSK health is determined by exposures accumulated across the life course. Key determinants include early motor development, physical activity and sport participation, occupational and caregiving demands, body composition, and comorbidities [1-3,17,24]. Repeated joint loading, particularly through high impact sport, heavy manual work, and kneeling or squatting, is associated with increased risk of osteoarthritis in later life [17,24]. Conversely, regular moderate physical activity and strength training can protect against falls, metabolic disease, and some forms of MSK pain [1-3,25,28-31].

The life course framework implies that preventive orthopedics must extend beyond perioperative care and post injury rehabilitation. It encompasses safe youth sport, healthy workplaces, early identification and management of osteoarthritis and low back pain, and fall and fracture prevention in older adults [3,7-9,14-16,22,23,25-31,36-39].

From Repair to Prevention

Orthopedic practice has historically emphasized surgical solutions. Yet many disabling MSK conditions arise from a sequence of potentially preventable events, such as ACL rupture followed by post traumatic knee osteoarthritis or low energy fragility fracture followed by subsequent fractures and loss of independence [17,25-27,32,33,60-63]. 

Recent scoping and conceptual reviews in chiropractic, osteopathy, physiotherapy, and sports medicine argue for a stronger focus on primary and secondary prevention, integrating biomechanical, psychosocial, and organizational factors [10-13]. Meyer and colleagues highlighted the importance of secondary prevention of chronic MSK pain by providing early, active, psychologically informed rehabilitation to individuals at risk of chronicity [11]. Draper-Rodi et al. proposed a prevention oriented framework that spans MSK care disciplines and explicitly distinguishes primary and secondary prevention strategies in MSK practice [12]. Holm-Jensen and colleagues emphasized early detection and early intervention for secondary prevention of sports injuries, further reinforcing the continuum of preventive opportunities [13].

Conceptual and scoping reviews in MSK health describe a preventive orthopedics paradigm that integrates risk stratification and tailored interventions, multifactorial programs combining exercise, ergonomics, education, and organizational strategies, and embedding interventions within real-world sport, workplace, and clinical systems supported by implementation science frameworks [7-13,43,44,46,47].

Current trends in preventive orthopedics

Sport and Physical Activity

Neuromuscular training and warm up programs: Injury prevention in youth and adult sport is one of the best developed areas of preventive orthopedics. Structured neuromuscular training (NMT) warm ups such as Federation of International Football Association (FIFA) 11 and FIFA 11 plus include progressive running, strength, balance, plyometric, and agility exercises performed as part of routine training [14-16,64-66].

Systematic reviews and meta-analyses show that these programs reduce overall injuries and importantly lower non-contact lower extremity injuries, including ACL and hamstring injuries, when implemented with adequate frequency and fidelity [14-16,64-66]. Thorborg et al. reported significant reductions in overall injury rate in football players using FIFA 11 or 11 plus compared with usual warm ups [14]. van Dyk et al. demonstrated that adding the Nordic hamstring exercise halves hamstring injury incidence across multiple sports [15]. Emery et al. concluded that NMT injury prevention strategies in youth sport reduce lower extremity injury risk, with incidence rate ratios of approximately 0.64 for lower extremity injuries in pooled analyses [66]. Robles-Palazón and colleagues used network meta-analysis to show that programs that integrate strength, balance, plyometrics, and technique appear most effective in youth team sport [16].

Umbrella reviews and implementation focused systematic reviews underscore that adherence and fidelity are key determinants of effectiveness [64,65]. Owoeye and co workers highlighted that high compliance with NMT programs leads to larger injury reductions in soccer [65]. Lutz et al. summarized best practices for dissemination and implementation of NMT warm ups in youth team sports, emphasizing coach education, integration into existing routines, and simple fidelity checks [64].

Relationship to long term joint health: ACL rupture and significant meniscal injury in youth and young adults are associated with a several fold increased risk of post traumatic knee osteoarthritis and earlier need for arthroplasty [17,65]. By reducing primary ACL injuries, NMT programs have the potential to lower downstream knee osteoarthritis incidence, providing a compelling example of primary and secondary prevention intersecting with long term joint preservation [14-17,60,65].

Representative evidence-based preventive interventions across the life course, spanning sport, community, fracture prevention, osteoarthritis, low back pain, overuse injuries, and workplace MSK programs, are summarized in Table 1.

**Table 1:** Selected evidence based preventive interventions in orthopaedics MSK: musculoskeletal; NMT: neuromuscular training; ACL: anterior cruciate ligament; FLS: fracture liaison service; USPSTF: U.S. Preventive Services Task Force; GLP-1: glucagon-like peptide-1

Population or domain	Intervention	Representative effect or summary	Key sources
Youth and adult team sport	NMT warm-ups (e.g., FIFA 11+), plyometrics, landing mechanics, Nordic hamstring exercise	Reductions in overall injuries and large relative reductions in ACL and hamstring injuries when implemented with adequate frequency and fidelity	[14-16,64-66]
Community-dwelling older adults	Exercise-based fall prevention with or without multifactorial assessment	Lower fall rates; USPSTF recommends individualized exercise programs for older adults at increased fall risk	[25,51,52]
Older adults with prior fragility fracture	FLS models including systematic identification, bone health assessment, treatment initiation, and follow-up	Increased osteoporosis testing and treatment; reduced refracture risk; cost-effective or cost-saving in multiple settings	[26,27,32-34,67,68]
Institutionalized older adults	Hip protectors	Reduced hip-fracture risk when worn; adherence and comfort are primary limitations	[69,70]
Knee and hip osteoarthritis	Weight loss plus exercise; guideline-concordant multimodal care	Improved pain and function; reduced knee joint loading with weight loss; possible delay of arthroplasty	[17-21,24]
Knee osteoarthritis in adults with obesity (emerging)	Pharmacologic weight management using GLP-1 receptor agonists (e.g., semaglutide)	Phase 3 trial evidence shows substantial weight loss, clinically meaningful reductions in knee pain, improved function, and reduced analgesic use; cohort data suggest possible disease-modifying effects; recent modeling shows potential cost-effectiveness	[56-58]
General and occupational populations with low back pain risk	Exercise alone or with education; individualized walking plus education; stratified secondary prevention	Reduced incident and recurrent low back pain; lower health-care use and absenteeism; reduced chronic pain risk in high-risk individuals	[11,12,28-31,71]
Overuse injuries in athletes and military personnel	Prefabricated foot orthoses; overpronation-controlling insoles; NMT and load management	Reduced overall injury and stress-fracture risk in some cohorts; NMT and insoles may reduce medial tibial stress syndrome	[16,22,23,66]
Workers in high-risk occupations	Ergonomic redesign, participatory ergonomics, conditioning and exercise programs	Reduced pain and MSK complaints; strongest evidence for health-care and dental professionals; benefits greater in multifactorial programs	[4-9,24,40]

Economic impact: Neuromuscular warm ups may also be economically attractive. In a pragmatic evaluation of a youth soccer injury prevention program, Lopatina and colleagues found that the neuromuscular training program reduced injuries and health care utilization and was cost effective from a health system perspective [35]. When broader societal costs and benefits, such as maintained sport participation and reduced time loss, are considered, the value of these programs is likely even greater [35,65].

Osteoarthritis prevention and lifestyle first care: Osteoarthritis is a leading cause of pain, disability, and joint replacement surgery. Contemporary guidelines from OARSI and the ACR emphasize exercise therapy, self management education, and weight reduction as foundational interventions, with pharmacologic and procedural options layered on as needed [17-19].

RCTs have demonstrated that intensive diet induced weight loss combined with exercise lowers knee joint loads and improves pain and function in overweight and obese adults with knee osteoarthritis [20,21]. Messier and colleagues showed that approximately 10 percent weight loss produced substantial reductions in knee compressive forces and symptom improvement [20]. Long term follow up suggests that sustained lifestyle changes can prolong benefits beyond the initial intervention period [21].

Primary prevention of osteoarthritis is more complex. Conceptual and epidemiologic work highlights challenges including the multifactorial nature of disease, difficulty identifying high-risk individuals early in life, and the long latency between risk exposure and clinical disease. Plausible preventive strategies include prevention of joint injuries, management of modifiable risk factors such as obesity and malalignment, and reduction of heavy occupational loading [17,24].

Pharmacologic weight management and GLP-1-based therapies: Pharmacologic weight management using GLP-1 receptor agonists and dual incretin agonists represents an emerging preventive strategy for knee osteoarthritis, particularly among individuals with obesity. Recent phase 3 evidence from the STepped Exercise Program for patients with Knee OsteoArthritis (STEP-Knee) trial shows that once-weekly semaglutide 2.4 mg produces substantial weight loss alongside clinically meaningful reductions in knee pain, improved physical function, and decreased analgesic use among adults with radiographically confirmed knee osteoarthritis [56]. Complementary cohort data further suggest that GLP-1 receptor agonists may slow knee osteoarthritis progression through combined mechanical load reduction and metabolic or anti-inflammatory pathways, as demonstrated in the Shanghai Osteoarthritis Cohort [57]. Despite growing evidence, GLP-1-based therapies remain underutilized in MSK preventive care, highlighting important implementation gaps. Emerging economic analyses indicate that semaglutide and tirzepatide may be cost-effective for patients with knee osteoarthritis and obesity, particularly when long-term disability reduction and cardiometabolic benefits are incorporated into models [58]. Integrating pharmacologic weight management with exercise, diet, and behavioral interventions may strengthen osteoarthritis prevention strategies, although long-term structural outcomes and equity considerations require further study.

Machine learning models based on radiographs, MRI, and clinical data have been developed to predict incident and progressive knee osteoarthritis [53-55]. These models may eventually assist in identifying high risk individuals for targeted lifestyle, biomechanical, or surgical interventions, though substantial work remains to validate and integrate such tools into clinical pathways.

Falls, Osteoporosis, and Fragility Fracture

Primary fall prevention: Falls and fall related fractures are major causes of morbidity, mortality, and loss of independence in older adults [1-3,25-27,69,70]. Multicomponent exercise programs that combine strength, balance, and gait training, sometimes with environmental assessments and medication review, reduce fall rates in community dwelling older adults [25,51,52]. The 2024 USPSTF recommends that clinicians selectively offer exercise interventions to prevent falls to community dwelling adults 65 years or older at increased risk, based on moderate certainty that these interventions confer a moderate net benefit [25].

The role of vitamin D supplementation in fracture prevention has been clarified by recent large trials. The VITamin D and OmegA-3 TriaL (VITAL) and related analyses found that supplementing generally healthy adults not selected for deficiency with vitamin D did not reduce fracture incidence compared with placebo [67]. Consequently, vitamin D is no longer recommended as a standalone primary fracture prevention strategy in this population.

Secondary fracture prevention and fracture liaison services (FLS): Secondary fracture prevention focuses on individuals who have already sustained a fragility fracture. FLS models identify fracture patients, assess bone health and fracture risk, initiate appropriate osteoporosis treatment, and provide long term follow up [26,27,32-34,67,68].

Systematic reviews and meta analyses demonstrate that FLS programs increase osteoporosis investigation and treatment initiation and reduce refracture rates [26,27]. Danazumi and colleagues concluded that FLS participation was associated with lower odds of subsequent fragility fractures and improved treatment uptake in adults aged 50 years and older [27].

Multiple economic evaluations support FLS as cost effective or cost saving. Li et al. used a Markov model with Dutch real-world data to show that FLS provides favorable incremental cost effectiveness ratios compared with no FLS [34]. Yong et al. evaluated a long standing FLS in Canada and found that it improved osteoporosis care and was cost effective over six years of service provision [32]. Saunders and co workers performed a cost utility analysis of the Ontario Fracture Screening and Prevention Program, a large FLS-like service, and reported favorable cost utility from the health system perspective [33]. Recent narrative and modeling work further highlights the broader economic benefits of fracture prevention, emphasizing that hip fracture prevention in particular yields substantial health and economic gains [68].

Hip protectors and institutional care: In nursing homes and other long term care settings, hip protectors provide a mechanical means of reducing impact forces during falls. Cochrane and umbrella reviews indicate that hip protectors probably reduce hip fracture risk in institutionalized older adults, although adherence is often low and device comfort and acceptability are key barriers [69,70]. These devices may thus be most useful as part of comprehensive fall and fracture prevention strategies supported by staff training and patient centered implementation approaches.

Osteoporosis screening: The 2025 USPSTF guideline recommends bone density screening for women aged ≥65 years and for younger postmenopausal women at elevated fracture risk, typically using DEXA [26]. DEXA is the gold standard for bone mineral density assessment, offering high precision, excellent test-retest reliability, low radiation exposure, and diagnostic thresholds aligned with World Health Organization (WHO) criteria (T-scores ≤ -2.5 indicating osteoporosis) [72]. Recent imaging reviews confirm that modern DEXA technology maintains strong accuracy and precision performance and remains central to fracture risk evaluation, with expanding applications in musculoskeletal assessment [72]. DEXA-derived Fracture Risk Assessment (FRAX) inputs further enhance fracture risk stratification. Screening strategies targeting treatment to individuals above established risk thresholds are repeatedly cost-effective or cost-saving in modeling analyses [26,32-34,68]. Integrating DEXA screening with fall-prevention and FLS programs represents a comprehensive preventive orthopedic strategy.

Occupational and workplace prevention: Work related MSK disorders are a major cause of disability, restricted duty, and compensation costs [4-9,24]. National surveillance systems such as National Institute for Occupational Safety and Health (NIOSH) and the National Safety Council document high rates of work related MSK injuries and illnesses, particularly in sectors with high physical demands and repetitive tasks [5,6].

Cochrane and other systematic reviews provide evidence that ergonomic interventions can reduce MSK symptoms and, in some contexts, injuries. In office workers, ergonomic interventions that involve workstation adjustments, posture training, and task redesign can lower discomfort and pain, although effects on specific disorders and absenteeism are moderate and heterogeneous [7]. Among dental professionals, ergonomic training, magnification loupes, and redesigned instruments are associated with reduced neck, back, and shoulder pain [5,8,40]. Nurses and other health care workers benefit from programs that combine manual handling training, mechanical lifting equipment, and organizational policies that limit high risk tasks; these multifaceted interventions can decrease MSK pain and time loss [9,40].

The WHO/International Labour Organization (ILO) joint estimates review by Hulshof et al. quantified the effect of occupational ergonomic risk factors on hip and knee osteoarthritis and other MSK diseases, finding increased risk associated with high force exertion, awkward postures, and vibration [24]. These data provide a further rationale for ergonomic and organizational interventions as part of preventive orthopedics.

A comprehensive systematic review by Albanesi and colleagues concluded that interventions to prevent and reduce work-related MSK injuries and pain among health care professionals are more effective when they are multifactorial, combining individual, task-specific, organizational, and environmental components [8,9].

Overuse Injury and Load Management

Overuse injuries are common in runners, military recruits, and athletes exposed to high training volumes. Systematic reviews suggest that prefabricated foot orthoses can reduce overall injury and stress fracture risk in military and athletic populations, whereas shock absorbing insoles alone have not shown consistent benefit [16,22,23]. Bonanno et al. reported that foot orthoses decreased overall injury and stress fracture incidence across several trials [22]. Marques et al. concluded that neuromuscular training and overpronation controlling insoles may reduce risk of medial tibial stress syndrome, though heterogeneity and risk of bias warrant caution [23].

The integrative scoping review identified multiple studies in military and tactical populations where modifications to training progression, load management, footwear, or bracing reduced lower limb soft tissue injuries [66,71]. Reviews of risk factors and prevention strategies in military personnel emphasize that both exercise and non exercise interventions, including footwear and equipment changes, can contribute to MSK injury prevention when properly integrated into training systems [66,71].

Low Back Pain

Low back pain is the leading contributor to MSK disability worldwide [1,2]. Exercise based interventions, alone or combined with education, reduce the incidence and recurrence of low back pain in general and occupational populations [28-31]. Shiri and colleagues found that exercise programs lower the risk of new episodes of low back pain compared with no intervention, especially when performed regularly [28]. Huang et al. concluded that both exercise alone and exercise plus education prevent low back pain episodes and reduce absenteeism [31].

The WalkBack trial showed that an individualized, progressive walking plus education program reduced recurrences of low back pain and decreased health care utilization compared with usual care in adults with a recent resolved episode [30]. A recent Cochrane review confirmed that exercise based programs can prevent nonspecific low back pain, although heterogeneity in exercise types limits firm conclusions about specific protocols [31].

Secondary prevention of chronic MSK pain more broadly has been synthesized by Meyer et al., who found that stratified programs combining education, reactivation, and cognitive behavioral approaches can reduce progression from acute or subacute pain to chronic pain [11]. Draper-Rodi and colleagues highlighted opportunities for MSK primary and secondary prevention within chiropractic, osteopathic, and physiotherapy practice, but also noted the paucity of high quality prevention focused trials [12].

Digital MSK programs are increasingly used for early management and secondary prevention. A randomized evaluation of a digital MSK acute care program reported improvements in pain and function and reductions in downstream health care utilization compared with usual care [71]. Such programs may help deliver evidence based education and exercise at scale, particularly when integrated with in person care pathways.

Pediatric Orthopedics

Developmental dysplasia of the hip (DDH) remains a key pediatric condition with a substantial preventive component. Universal ultrasound screening detects more dysplastic hips and increases early nonoperative treatment compared with selective strategies but has not consistently reduced late detection or operative treatment in all settings [60-62]. Shorter et al. showed that universal ultrasound screening detects more abnormalities but may increase overtreatment [60]. Westacott and colleagues, and more recently Cheok et al., reported that universal screening may lower the incidence of late diagnosis in some programs [61,62]. Biedermann and Eastwood discussed the trade offs between universal and selective screening and emphasized the critical importance of high quality clinical examination and selective ultrasound for infants with risk factors [63].

Beyond DDH, pediatric preventive orthopedics emphasizes the development of fundamental movement skills and safe participation in physical activity to reduce injury risk and promote lifelong MSK health [3,10-13].

Benefits of preventive strategies in orthopedic care

Clinical Outcomes

Across domains, preventive orthopedic strategies deliver meaningful benefits on clinical outcomes. In youth and adult sport, NMT warm ups and targeted eccentric strengthening reduce soft tissue and ligamentous injuries, especially ACL and hamstring injuries [14-16,64-66]. By reducing serious knee injuries, these programs may also lower the risk of later osteoarthritis and need for joint replacement [17,65].

Lifestyle first osteoarthritis care improves pain, function, and quality of life in knee and hip osteoarthritis and can reduce joint loading, potentially slowing structural deterioration [17-21]. Fall prevention programs lower falls in community dwelling older adults [25,51,52], while FLS programs improve osteoporosis treatment and reduce refractures [26,27,32-34,67,68]. Hip protectors reduce hip fractures in institutional settings among adherent users [69,70].

Exercise plus education programs prevent new episodes and recurrences of low back pain [28-31] and may reduce the progression to chronic pain when applied early and in a stratified fashion [11,12]. Overuse injury prevention interventions, including foot orthoses and load management, decrease certain lower limb overuse injuries and stress fractures, particularly in military and high volume athletic settings [16,22,23,66].

Prehabilitation before orthopedic surgery improves preoperative function and early postoperative outcomes. Systematic reviews by Punnoose et al., Gränicher et al., Konnyu et al., and others have shown that prehabilitation improves strength, pain, and function before and within the first year after total knee arthroplasty and other orthopedic procedures [36-38]. A recent randomized trial in high risk total knee arthroplasty patients reported improved short term function with targeted prehabilitation [39]. A newer meta-analysis suggests that prehabilitation can relieve pain after knee arthroplasty, particularly in the early postoperative period [40].

Health System and Economic Value

Preventive orthopedics also has important system level and economic implications. Hip fractures, joint replacements, and chronic MSK pain generate significant direct medical costs and indirect costs from loss of productivity and caregiving [1-6,32-34,68]. Economic evaluations increasingly show that key preventive programs provide good value.

FLS programs have been repeatedly shown to be cost effective or cost saving in various health systems [27,32-34,68]. Markov models and real world evaluations indicate that modest per patient investments in FLS are offset by reductions in secondary fractures and associated health care costs, resulting in favorable incremental cost effectiveness ratios [32-34].

Neuromuscular injury prevention programs in youth soccer have been shown to be cost effective, with lower injury-related costs and preserved sport participation outweighing program costs [35]. Economic analyses of fracture prevention strategies highlight that hip fracture prevention and comprehensive fracture prevention bundles (fall prevention, osteoporosis screening, FLS) deliver particularly favorable economic returns [26,32-34,68].

Workplace MSK prevention can reduce sickness absence, workers compensation claims, and productivity losses, although the economic evidence is more heterogeneous and context dependent [4-9,24].

Patient-Centered Outcomes

Preventive orthopedic interventions also affect outcomes that patients value, such as pain relief, preservation of independence, ability to maintain employment, and participation in meaningful activities. Prehabilitation, fall prevention, and chronic pain prevention programs commonly report improvements in health related quality of life and patient satisfaction, in addition to objective clinical measures [11,25-31,36-40,71]. Digital MSK programs may enhance patient centeredness by enabling flexible, home-based participation and tailored feedback [48-52,71].

Challenges and barriers to implementation

Evidence Heterogeneity and Generalizability

The preventive orthopedics evidence base spans diverse populations, interventions, and outcomes. Trials differ in design, adherence, duration, and co-interventions, which complicates synthesis and generalization [7-9,11-16,22-31,36-39,66]. Many studies involve relatively homogeneous cohorts, such as young athletes or specific occupational groups, limiting applicability to broader, more diverse populations.

In some domains, such as primary prevention of osteoarthritis, secondary prevention of sports injuries, and workplace MSK programs in non-health care sectors, evidence remains limited or of moderate quality [9,11-13,24]. This uncertainty hinders guideline development and large scale implementation.

Adherence and Fidelity

The effectiveness of exercise-based and multifactorial interventions depends heavily on adherence and fidelity. NMT programs yield larger injury reductions when teams perform the full program at the recommended frequency and intensity [14-16,64-66]. However, real-world studies show that coaches often shorten warm ups or omit key components due to time constraints or competing priorities [64,65].

Common implementation barriers and pragmatic solutions, mapped to real-world examples and key implementation frameworks, are outlined in Table 2.

**Table 2:** Common barriers to preventive orthopedics and pragmatic solutions NMT: neuromuscular training; RE-AIM: Reach, Effectiveness, Adoption, Implementation, Maintenance; CFIR: Consolidated Framework for Implementation Research; FLS: fracture liaison service; TIDieR: Template for Intervention Description and Replication; SANRA: Scale for the Assessment of Narrative Review Articles; ML/AI: Machine learning/Artificial intelligence; OA: Osteoarthritis; GLP-1: glucagon-like peptide 1; EMR: Electronic medical record;

Barrier	Illustrative context	Pragmatic solution(s) and frameworks	Key sources
Low adherence and diluted fidelity	Coaches abbreviate NMT warm-ups; older adults discontinue fall-prevention exercise; limited adherence to prehabilitation; low engagement with digital MSK tools	Simplify and standardize protocols; embed into existing routines; coach/clinician training; fidelity checks; digital reminders; evaluate reach and maintenance using RE-AIM	[14-16,24,25,30,36-40,47,50-52,64-66,71]
Workforce and time constraints	Limited time/space for supervised exercise, ergonomic training, or prehabilitation; competing workflow demands	Develop brief or hybrid delivery models; integrate exercise into work routines; use telehealth; apply CFIR to identify contextual barriers and facilitators	[4-9,24,36-40,46]
Equity and digital divide	Reduced access to supervised programs in rural/low-resource settings; limited Internet/device access for digital MSK or fall-risk tools	Low-cost community delivery; leverage primary care/community health workers; SMS/phone-based supports; monitor digital access; co-design with underserved groups	[3-9,25-27,48-52,71]
Outcome heterogeneity and poor reporting	Variable injury definitions; inconsistent outcome measures; incomplete description of intervention components	Use core outcome sets; standardize injury incidence/exposure reporting; apply TIDieR and TIDieR-Rehab; use SANRA for transparent narrative synthesis	[7-9,11,14-16,22-24,28-31,36-39,41,43,44,66]
Care gaps after fragility fracture	Many patients do not receive osteoporosis screening, medications, or FLS enrollment after a fracture	Implement coordinated FLS models; automated patient identification; registry integration; audit/feedback cycles; evaluate clinical and economic outcomes	[26,27,32-34,67-69]
Limited integration of emerging pharmacologic prevention (GLP-1)	GLP-1 receptor agonists show significant weight reduction, knee-pain improvement, and potential disease-modifying effects in OA, yet remain rarely incorporated into preventive orthopedic pathways	Incorporate GLP-1 therapy into multimodal OA prevention; update clinical pathways; assess long-term cost-effectiveness; address prescribing and insurance barriers	[56-58]
Implementation challenges for ML/AI and smartphone fall-risk tools	AI models and smartphone-based gait analysis validated in 2024–2025 studies but remain underutilized; concerns about workflow integration, liability, accuracy in diverse populations, and digital inequity	Pilot hybrid effectiveness-implementation trials; integrate into EMR; ensure external validation; monitor unintended inequities; apply RE-AIM/CFIR for implementation mapping	[48-55]

Similarly, adherence to fall prevention exercise declines over time, especially in frailer older adults or those with cognitive impairment [25,50-52]. Prehabilitation uptake can be limited by time before surgery, transportation barriers, and patient fatigue [36-40]. Digital programs can improve access but may encounter engagement challenges among individuals with low digital literacy [50-52,71].

Equity and Access

Preventive services are not equitably distributed. Rural populations, individuals in low resource settings, and workers in precarious employment often have less access to supervised exercise, physiotherapy, ergonomic support, or FLS [3-9,25-27,51,52]. Mobile health and digital tools may mitigate some barriers but introduce others, including the digital divide related to device ownership, connectivity, and digital literacy [48-52,71].

Scoping and implementation reviews highlight the importance of participatory design, involvement of local stakeholders, and adaptation to context to ensure that preventive programs are acceptable, feasible, and accessible in diverse populations [9-13].

Measurement and Reporting

Heterogeneity in outcome measures and incomplete reporting of interventions hinder synthesis and replication. Trials use a wide range of injury definitions, pain scales, functional measures, and resource use metrics, making it difficult to compare results or pool data [7-9,11,14-16,22-24,28-31,36-39,66].

Reporting guidelines such as TIDieR and TIDieR-Rehab provide structured templates for describing complex interventions and their delivery, but adherence remains variable [43,44]. Wider use of these checklists, along with condition specific core outcome sets, would strengthen the preventive orthopedics evidence base and facilitate translation into practice [41,43,44].

Implementation Capacity and Frameworks

Relatively few preventive orthopedics studies explicitly employ implementation science frameworks. CFIR offers constructs to assess intervention characteristics, inner and outer settings, characteristics of individuals, and implementation processes [46]. RE-AIM encourages evaluation of reach, effectiveness, adoption, implementation, and maintenance [45].

Scoping and systematic reviews of MSK programs in military and workplace settings suggest that adoption, reach, fidelity, and sustainability are often under measured, leaving important knowledge gaps about how to successfully scale interventions [9-11,24,64,66]. Greater use of hybrid effectiveness implementation designs and embedded implementation evaluation is needed [10,45-47].

Future directions and research priorities

Operationalizing Life Course MSK Prevention

Preventive orthopedics should be systematically embedded across life stages. In youth, policy and governing body support are needed to make NMT warm-ups standard in school and club sport, accompanied by coach education and simple fidelity monitoring [14-16,35,64-66]. For adults in high risk occupations, multifactorial workplace programs that combine ergonomic redesign, participatory ergonomics, and conditioning should be scaled and rigorously evaluated [4-9,24,40].

For older adults, combining fall prevention exercise, osteoporosis screening, and FLS offers a comprehensive fracture prevention strategy with strong clinical and economic rationale [25-27,32-34,67,68].

Pragmatic- and Equity-Focused Trials

Pragmatic randomized trials and hybrid effectiveness implementation designs are needed to understand how preventive orthopedic interventions perform in real-world conditions and how best to enhance adoption and sustainability [10-13,36-39,46,47,64]. Cluster randomized or stepped wedge designs are particularly appropriate for team-based, workplace, or facility-level interventions.

Equity should be a prespecified focus, with subgroup analyses by sex, age, race and ethnicity, socioeconomic status, geography, and digital access. Scoping reviews of MSK prevention note sparse evidence in many underserved populations and call for greater attention to social determinants of MSK health [3,10-13].

Illustrative research priorities that operationalize these needs across sport, fracture prevention, digital technologies, prehabilitation, and prevention frameworks are summarized in Table 3.

**Table 3:** Illustrative research priorities in preventive orthopedics NMT: neuromuscular training; CFIR: Consolidated Framework for Implementation Research; RE-AIM: Reach, Effectiveness, Adoption, Implementation, Maintenance; FLS: fracture liaison service; MSK: musculoskeletal; LOS: Length of stay; OA: Osteoarthritis; GLP-1: Glucagon-like peptide 1; RCT: randomized controlled trial.

Priority question	Suggested design	Key outcomes and notes	Key sources
How can adoption and sustainability of NMT warm-ups in youth sport be maximized at scale?	Cluster-randomized or stepped-wedge trials with embedded implementation evaluation (hybrid type II or III) using CFIR and RE-AIM	Injury incidence; program fidelity; reach, adoption, maintenance; cost per injury prevented; qualitative coach/athlete feedback	[14-16, 35,45-47,64-66]
Which FLS components most influence refracture reduction and cost-effectiveness across health systems?	Multicenter registries; hybrid effectiveness–implementation trials; Markov/state-transition economic modeling	Refracture rates; time to treatment initiation; treatment adherence; mortality; incremental cost-effectiveness ratios; equity of access	[26,27,32-34,68]
Can smartphone-based gait analysis and wearable fall-risk tools improve outcomes when embedded in primary care and community programs?	Pragmatic randomized or stepped-wedge trials with adaptive algorithms; equity-focused implementation analyses	Falls and injurious falls; uptake/adherence; usability/acceptability; false-positive and false-negative rates; digital access metrics; cost-effectiveness	[48-52]
Which patient phenotypes benefit most from prehabilitation before arthroplasty and spine surgery, and what is the optimal dose?	Risk-stratified randomized trials; dose–response studies; hybrid in-person + tele-prehab evaluations	Pre-/postoperative function; pain; LOS; complications; return to work/sport; cost-utility; phenotype-specific response	[36-40]
How can primary, secondary, and tertiary prevention be operationalized and standardized across MSK care?	Conceptual and scoping reviews; Delphi expert consensus; observational cohorts incorporating risk stratification	Clear prevention definitions; validated risk tools; consensus on core prevention outcomes and reporting standards	[10-13,41, 59]
How accurate and clinically useful are machine-learning models for identifying individuals at high risk of incident or progressive knee OA?	External validation cohorts; prospective prediction-impact studies; workflow-integration trials	Model discrimination/calibration; clinical decision impact; interpretability; effect on referral patterns; downstream outcomes	[53-55]
Do GLP-1 receptor agonists meaningfully reduce long-term OA progression or delay arthroplasty in individuals with obesity?	Longitudinal RCT extensions; pragmatic trials; comparative-effectiveness studies vs lifestyle-only care	Structural progression (radiographic/MRI); pain and function trajectories; weight change; need for injections or surgery; cost-effectiveness; equity in access	[56-58]

Refining Fracture Prevention and FLS Models

Despite strong evidence, FLS coverage remains incomplete in many health systems. Research priorities include identifying the most influential components of FLS, optimizing referral and follow-up pathways, leveraging telehealth and digital tools for follow up, and ensuring implementation in low- and middle-income settings [26,27,32-34,68].

Further refinement of osteoporosis screening thresholds and treatment algorithms, including alignment with new therapies, is also needed to maximize fracture reduction while maintaining cost effectiveness [26,32-34,68].

Digital and Data-Driven Preventive Orthopedics

Wearables and mobile technologies can quantify workload, balance, and movement patterns, enabling early detection of injury risk and more individualized prevention strategies [48,49]. Smartphone-based gait and mobility assessments, including multi-sensor gait analysis and digital Timed-Up-and-Go tests, demonstrate strong discriminative accuracy and high test-retest reliability for identifying older adults at elevated fall risk, including those with cognitive impairment, and are increasingly integrated into community fall-prevention programs [50-52].

Deep learning models applied to radiographs and MRI can now automate early detection and grading of knee osteoarthritis and predict incident or progressive disease with high diagnostic accuracy, as shown across recent validation cohorts [53-55]. These tools have the potential to support targeted prevention and timely intervention if integrated into clinical workflows without widening existing equity gaps.

Virtual and hybrid MSK care programs further expand access to prevention by delivering education, exercise therapy, and remote monitoring. Randomized evaluations of digital MSK platforms demonstrate improvements in pain, function, and reductions in downstream health-care utilization, supporting their role in scalable secondary and tertiary prevention [71].

Advancing Prehabilitation Science

Prehabilitation is a prototypical tertiary preventive intervention. Future research should clarify optimal dose, content, and timing of prehabilitation across procedures and risk phenotypes [36-40]. Risk stratified approaches that focus resources on patients most likely to benefit, such as those with frailty, obesity, or poor baseline function, should be developed and tested [36-40].

Cost effectiveness analyses of prehabilitation in routine care and tele-prehabilitation models will be important to inform health system decisions [36-40]. Integration with digital monitoring and wearable technologies may enhance adherence and enable personalization.

Conceptual and Methodological Development

Conceptual reviews underline the need for clearer definitions and metrics for MSK primary, secondary, and tertiary prevention and for better alignment between prevention frameworks and orthopedic practice [10-13]. Adoption of quality tools such as SANRA for narrative reviews, broader use of TIDieR and TIDieR-Rehab, and development of MSK prevention-specific core outcome sets will help improve the quality, transparency, and utility of future research [41,43,44].

## Conclusions

Preventive orthopedics reframes MSK care from a reactive, procedure-centered model to a proactive, life course strategy aimed at avoiding first injuries, preventing recurrence, and minimizing long-term disability. Strong evidence supports core elements, such as neuromuscular training in youth sport, lifestyle first osteoarthritis care, fall and fracture prevention, workplace ergonomic programs, and perioperative prehabilitation, each demonstrating meaningful improvements in pain, function, and quality of life, alongside favorable economic value.

Despite this progress, significant gaps remain in implementation, fidelity, and equitable access. The next advancement in preventive orthopedics is not more proof of efficacy, but sustainable translation into routine practice. Embedding prevention across clinical pathways, sport and workplace systems, and digital care models, guided by implementation science and equity principles, offers a critical opportunity to reduce the growing global burden of MSK disability and support healthier aging for all.
